# Supplementary material for: Comparative Pathogenesis, Genomics and Phylogeography of Mousepox
Source: Viruses. 2021 Jun 15;13(6):1146. doi: 10.3390/v13061146 (PMC8232671; doi:10.3390/v13061146)
Supplement: Supplementary file 1 [file viruses-13-01146-s001.zip › Supplentary files/Supplementary data_R1_NEW.pdf]

Supplementary data

Figure S1

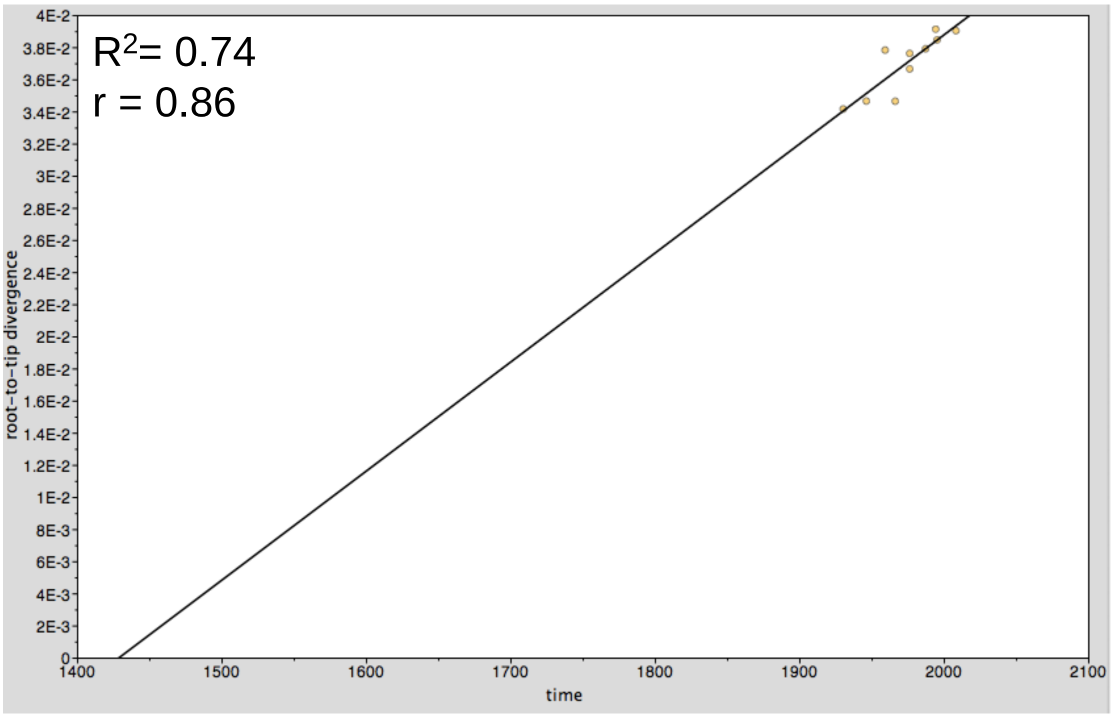

Figure S1. Regression analysis of temporal resolution of ECTV sequence dataset. The plot represents linear regression of root-to-tip genetic distance within the ML phylogeny against sampling time for each taxa. Temporal resolution was assessed using the slope of the regression, with positive slope indicating sufficient temporal signal. Correlation coefficient “r” and “R2” are reported.

Tables

Table S1. Survival for Figure 1.

Isolate	pfu						
	**1**	**10**	**10^2**	**10^3**	**10^4**	**10^5**	**10^6**
ECTV-M	0/5	0/5	0/5				
ECTV-I					5/5	5/5	4/5
ECTV-MH					5/5	5/5	5/5
ECTV-HE					5/5	5/5	5/5
ECTV-H	3/10	6/10	4/10	4/5			
ECTV-M1	5/10	2/10	0/5	0/5			
ECTV-M4	5/5	4/5	5/5	7/10	0/5	0/5	
ECTV-M5	5/5	4/5	4/10	1 /10			
ECTV-MK	5/5	3/10	2/10	0/5			

Table S2. Survival of DBA/2 mice after s. c. infection in the footpad with ECTV isolates.

Isolate	**0.01**	**0.1**	**1**	**10**	**10^2**	**10^3**
ECTV-M	0/3	1/3	3/3	3/3	2/3	3/3
ECTV-N	0/3	1/3	3/3	3/3	2/3	3/3
ECTV-H	0/3	1/3	3/3	3/3	2/3	1/3
ECTV-I	0/3	0/3	0/3	0/3	0/3	0/3

Table S3. Comparative analysis of the proteins encoded by ECTV-Moscow, ECTV-Naval and the ECTVs from this study. Excel file.
